# Limiting worker exposure to highly pathogenic avian influenza a (H5N1): a repeat survey at a rendering plant processing infected poultry carcasses in the UK

**DOI:** 10.1186/1471-2458-11-626

**Published:** 2011-08-05

**Authors:** Nicol Coetzee, Obaghe Edeghere, Musarrat Afza, Harsh V Duggal

**Affiliations:** 1 Health Protection Agency, West Midlands North, Crooked Bridge Road, Stafford, ST16 3NE, UK; 2 Health Protection Agency, Regional Epidemiology Unit, 5 St Phillips Place, Birmingham, B3 2PW, UK

## Abstract

**Background:**

Current occupational and public health guidance does not distinguish between rendering plant workers and cullers/poultry workers in terms of infection risk in their respective roles during highly pathogenic avian influenza poultry outbreaks. We describe an operational approach to human health risk assessment decision making at a large rendering plant processing poultry carcasses stemming from two separate highly pathogenic avian influenza A (H5N1) outbreaks in England during 2007.

**Methods:**

During the first incident a uniform approach assigned equal exposure risk to all rendering workers in or near the production line. A task based exposure assessment approach was adopted during the second incident based on a hierarchy of occupational activities and potential for infection exposure. Workers assessed as being at risk of infection were offered personal protective equipment; pre-exposure antiviral prophylaxis; seasonal influenza immunisation; hygiene advice; and health monitoring. A repeat survey design was employed to compare the two risk assessment approaches, with allocation of antiviral prophylaxis as the main outcome variable.

**Results:**

Task based exposure assessment during the second incident reduced the number of workers assessed at risk of infection from 72 to 55 (24% reduction) when compared to the first incident. No cases of influenza like illness were reported in workers during both incidents.

**Conclusions:**

Task based exposure assessment informs a proportionate public health response in rendering plant workers during highly pathogenic avian influenza H5N1 outbreaks, and reduces reliance on extensive antiviral prophylaxis.

## Background

Avian Influenza outbreaks on commercial poultry farms have recently been identified in several European countries including the United Kingdom (UK) [1-4]. The public health response to these incidents is based on guidance tailored to the poultry industry. Pre-exposure antiviral prophylaxis serves as an important intervention even though there is limited evidence of additional benefit above that provided by physical measures (masks, gowns, gloves and handwashing) in reducing the spread of respiratory viruses [5]. Additional public health measures include personal protective equipment, seasonal influenza vaccines (aimed at preventing viral re-assortment during co-infection) and health monitoring of the affected population [6,7]. The main occupational groups covered by this guidance include poultry workers, cullers and others who have close or direct contact with infected live poultry and their environment during culling operations [8].

Highly pathogenic avian influenza (HPAI) H5N1 outbreaks require extensive culling of infected birds yielding high numbers of carcasses to be safely disposed of at industrial animal tissue rendering plants - the preferred method of disposal in the United Kingdom [9]. Rendering is a process that converts waste animal tissue into stable materials like tallow and protein meal. Rendering plant workers are not exposed to live infected poultry and likely to be at lower risk of HPAI H5N1 infection compared to commercial poultry workers and those involved in culling operations (catching, killing, and evisceration of birds). There is some transmission risk in rendering workers because influenza viruses survive for some days in carcasses, body fluids and some contaminated surfaces [10,11]. The transportation of infected carcases for off-farm rendering has been implicated in avian influenza transmission between commercial poultry farms [12]. However, in contrast to highly exposed poultry workers and cullers, to date there have been no reports of HPAI infection in rendering plant workers [13,14].

Workers at rendering plants are not specifically covered by the existing health protection guidance for poultry workers, thus requiring context-specific human health risk assessments during rendering operations involving HPAI infected carcasses [6,8,15].

In order to inform future public health practice and targeted prophylactic antiviral prescribing, we describe our experience in refining the exposure risk assessment for workers at an industrial rendering plant processing infected poultry carcasses following culling operations during two HPAI H5N1 outbreaks in Suffolk, England in February and November 2007 [3,4]. These incidents resulted in approximately 160,000 carcasses being rendered in February 2007 (Incident I) and 100,000 in November 2007 (Incident II) [3,4].

## Methods

### Study population

The rendering plant is situated in the West Midlands (180 miles from the affected farms in Suffolk), and employs a constant workforce complement of 135 shift workers delivering a 24 hour operation. Two of five production lines are dedicated to processing infected animal tissue. The main occupational categories are administration, production management, road (long distance) transport drivers, on-site shunter drivers, raw material operatives, general maintenance staff, and electricians. During both incidents 22 animal health officers were drafted in to supervise the rendering process giving an overall plant workforce complement of 157.

Long distance truck drivers transported infected carcasses in sealed trailers from the outbreak sites to the rendering plant entrance, but did not participate in loading or unloading carcasses. On-site shunter drivers transport the full trailers to the start of the rendering production lines where the carcasses are mechanically unloaded directly into hoppers. Raw material operatives manually scoop up any spillage (carcasses and tissues) and the shunter drivers then wash down the trailers and reception area using high pressure water jets. The ensuing production process is automated and involves crushing the carcasses, followed by high temperature heating (steam cooker), and finally the separation of residue into sterile fat (tallow) and solids (meat and bone meal). It requires constant oversight by raw material operatives and if repairs are needed, the production process is halted and general maintenance personnel or electricians undertake repairs which may involve working in confined spaces contaminated with raw material.

### Human health risk assessment

During both incidents, the local health protection unit used direct observation to assess the potential for worker exposure to infection and advised on prevention measures prior to exposure. Our approach to human health risk assessment was adapted and refined over the two incidents. A "uniform" exposure risk assessment used during incident I was replaced by a "targeted" task based exposure assessment method during incident II.

During incident I the health protection team had only a few hours advanced notice before delivery of the first consignment of poultry carcasses to the plant. For logistical reasons, a uniform "infected premises" risk assessment approach was adopted with clearly defined movement restriction zones similar to that used at the poultry farm outbreak sites [16]. The restriction zones on the plant premises included the road used to transport carcasses and the two production lines. All staff entering restriction zones (irrespective of their occupational category) were assessed as having uniform exposure risk without considering differences in individual occupational functions or tasks.

During incident II our prior experience informed the development of a task based exposure assessment. Work processes were studied by direct observation and categorised into risk levels based on actual or potential exposure to infected carcasses and contaminated surfaces. Four occupational risk categories (A to D) were identified and interventions tailored accordingly (Table 1).

**Table 1:** Risk categories and recommended preventive interventions in rendering plant workers potentially exposed to H5N1 infected poultry carcasses as applied during Incident II.

Risk Group	Exposure category	Intervention provided	Occupational group
A. High risk	Direct contact - handling infected raw material		
			
	Droplet or aerosol - contact with infected liquids or aerosol generated by high pressure water clearing within confined space	PPE, antiviral prophylaxis (oseltamivir), seasonal influenza vaccine	Raw material operatives; shunter drivers;
		
B. Medium risk	Indirect contact - handling/touching contaminated surfaces; work in confined production line spaces		General maintenance personnel

C. Low risk	Working in close proximity (within 1 metre) to infected dead poultry (but no exposure as described above)	PPE, seasonal influenza vaccine, with/without antiviral prophylaxis (based on individual assessment of exposure risk)	Production managers; general manager; electricians; duty manager; animal health officers

D. Negligible risk	Not sharing the same confined space with infected dead poultry, and no direct, indirect, droplet or aerosol contact	None	Managerial staff; administrative staff; long distance truck drivers

PPE (personal protective equipment): filter face piece, protection level 3 (FFP3) respirator masks, eye goggles, splash proof overalls, gloves, boots, and hard hats.

### Protective interventions

Rendering workers assessed to be at risk of infection exposure during the two incidents were offered pre-exposure antiviral prophylaxis (oseltamivir 75 mg per day for minimum of 10 days). The rendering of infected carcasses lasted on average 5 days and based on individual worker shift patterns, prophylaxis was extended for 7 days after last exposure. The allocation of antiviral prophylaxis therefore serves as a proxy measure for workers assessed as being at risk of infection. At risk workers were also offered seasonal influenza immunisation (if unvaccinated); personal protective equipment (PPE) (consisting of filter face piece protection level 3 (FFP3) respirator masks, eye goggles, splash proof overalls, gloves, boots, and hard hats - according to health and safety recommendations); instruction on hygiene measures and hand washing; and asked to report influenza-like illness symptoms to their general practitioner for up to two weeks after last exposure [6,8,15]. In addition, the study team wrote to the general practitioners of all workers asking them to report by telephone any cases of influenza-like illness during this period.

### Study design and analysis

We undertook a repeat descriptive survey comparing worker risk assessment approaches across the two incidents. Workers consented to participation and confidential records were kept by the plant management. During both incidents, records were kept of the entire workforce detailing their occupational category, exposure risk categorisation, and subsequent allocation of antiviral prophylaxis as the main outcome variable. Data on allocation of seasonal influenza vaccination was also collected. The analysed data is presented as counts and proportions by occupational risk category, and absolute differences between proportions in the two incidents with no statistical test undertaken to determine the significance of the difference between the observed proportions.

### Ethics approval

Not required.

## Results

During both incidents all workers assessed as having potential for infection exposure accepted antiviral prophylaxis. No workers were exposed to infectious material prior to receiving antiviral prophylaxis. The number of workers assessed as being at risk of infection (offered antiviral drugs) decreased from 72 (46%) during incident I to 55 (35%) during incident II. This reduction is apparent in occupational risk groups A, B, and D (Table 2). In occupational risk group C, the proportion allocated antiviral prophylaxis increased during incident II with an absolute difference of 17% between incidents (Table 2).

**Table 2:** Administration of pre-exposure antiviral prophylaxis by occupational risk category comparing different risk assessment approaches used in incident I and incident II.

Occupational risk group	Total worker population	At risk population in receipt of intervention				Absolute difference
	N	Incident I		Incident II		P2 - P1
		n	% (P1)	n	% (P2)	
A (high risk)	37	17	46	15	41	-5
B (medium risk)	17	12	71	9	53	-18
C (low risk)	35	18	51	24	69	17
D (negligible risk)	68	25	37	7	10	-26

TOTAL	157	72	46	55	35	-11

During incident I, 60 (83%) at-risk workers accepted seasonal influenza immunisation; 4 (5%) had prior immunisation; and 8 (11%) declined immunisation. During incident II, 45 (81%) at risk workers accepted seasonal influenza immunisation with the remainder declining or having had prior immunisation. No cases of influenza-like illness were reported to general practitioners in the two weeks following last potential exposure in both incidents.

## Discussion

The use of a task based exposure assessment approach during incident II resulted in 17 (24%) fewer workers categorised as being at risk and prescribed oseltamivir prophylaxis. This approach allowed the exclusion of a significant number of workers in the very low risk category D group (particularly long distance truck drivers and administrative staff) and limiting the number in high risk categories (A & B). Due to a communication error, seven category D workers did receive antiviral prophylaxis (but not PPE) at the onset of incident II. The increase in the proportion of category C workers receiving the interventions (including antiviral prophylaxis and PPE) during incident II is due to a precautionary increase in supervisory staff (duty and production managers) during protracted shift work. The reduction in workers requiring seasonal influenza vaccine during incident II is only in part due to task based exposure assessment, as some would have had prior immunisation.

The findings from an exposure risk assessment of carcass disposal options appears to suggest that exposure to and the risk of H1N1 infection is likely to be low in the latter stages of the carcass disposal process such as during the rendering of killed poultry[17].

However, specific guidance on risk assessment and management of rendering workers is not available and related guidance is conflicting. For example, the European Centre for Disease Prevention and Control guidance classifies rendering workers under the "direct contact" group [18]. Occupational health and safety guidance in Northern Ireland excludes rendering workers from the at-risk group as they are judged not to have "direct contact with sick, dead or dying infected birds or bird products" [19]. The existing Health Protection Agency guidance also does not specifically cover rendering workers and the application of the guidance to this population is open to interpretation [5]. In addition, in the UK, rendering plant workers are specifically excluded from guidance recommending annual pre-exposure seasonal influenza immunisation of poultry workers [20].

Although the uniform risk assessment approach (using the "infected premises" principle) adopted in "Incident I" can be implemented rapidly and easily enforced, it lacks the specificity required when advising anxious workers on the need for antiviral prophylaxis. The uniform approach clearly has merits during culling operations on poultry farms with large numbers of temporary workers drafted in at short notice. By contrast, the controlled rendering plant environment is well suited to a task based exposure assessment approach due to the clear demarcation of work areas, operational processes and associated occupational functions. Adopting task based worker exposure assessment prior to HPAI outbreaks has application to all types of poultry industry workers as a means to ensuring a risk based and proportionate response to such situations.

The safety and efficacy of neuraminidase inhibitors has already been demonstrated extensively in studies of adults taking chemoprophylaxis following exposure to the seasonal influenza virus [21,22]. However, there is a paucity of research evidence on the safety and efficacy of these agents against H5N1 particularly when used for short and longer-term prophylaxis particularly in low-risk occupational groups [23]. Although we did not monitor compliance and measure the incidence of adverse events in our study population, it is possible that minor adverse events went undetected.

This study proposes improvements in the risk assessment methodology based on an understanding of the occupational and industrial process and was not designed to estimate the actual level of infection risk in rendering workers. The total absence of reported influenza like illness in workers up to 14 days after exposure to the rendering process, despite adopting a task based exposure assessment approach, is encouraging. Although it is possible that very mild cases may not have been detected, we have no reason to believe there was under-ascertainment of influenza like illness cases by general practitioners serving this workforce. A definitive evaluation of the potential for avian-to-human transmission of H5N1 in rendering workers would necessitate undertaking a cohort study comparing paired serum samples in all occupational categories over both incidents. Although there is evidence of potential for transmission in poultry workers and cullers, their exposure risk is significantly higher than rendering workers [13,14]. This study also does not measure the added benefit of antiviral prophylaxis in comparison to barrier methods in preventing infection.

Even though the full workforce complement (by category and person-hours worked) at the plant remained largely unchanged, there were seasonal workers employed during the two incidents. This means that we cannot fully assume that the study population represents paired groups with observations taken from the same individuals during the two incidents, an assumption that is required to undertake appropriate statistical tests such as a McNemars test. We would have analysed the sub-set of the population with paired observations, but the heterogeneous nature of the study population (paired and unpaired) precluded our ability to link the observations recorded during the two incidents to specific individuals so as to identify discordant pairs.

The perception that the risk of infection and transmission is uniformly low in rendering workers has not been validated, particularly in terms of the higher risk occupational categories. Work to measure task based H5N1 exposure risk and biological outcomes (measures of infection and seroconversion) should be undertaken in future.

## Conclusions

The use of a task based exposure assessment approach was practical to implement and has relevance for the protection of all workers involved in rendering HPAI infected poultry carcasses. Evidence based guidance that focuses on the highest risk occupational groups will help contain costs, and limit the extent of reliance on antiviral prophylaxis. In addition, this may have benefits in reducing the risk of adverse events and the development of drug resistance.

## Competing interests

The authors declare that they have no competing interests.

## Authors' contributions

NC conceived of the study, participated in its design, data collection, analysis and interpretation, and drafting of the manuscript. OE contributed to study design, data analysis, interpretation, and drafting of the manuscript. MA contributed to analysis, interpretation of data and drafting the manuscript. HVD contributed to analysis, interpretation of data and critical revision of the manuscript. All authors read and approved the final manuscript.

## Pre-publication history

The pre-publication history for this paper can be accessed here:

http://www.biomedcentral.com/1471-2458/11/626/prepub

## References

[B1] Puzelli S Di Trani L Fabiani C Campitelli L De Marco MA Capua I Aguilera JF Zambon M Donatelli I Serological analysis of serum samples from humans exposed to avian H7 influenza viruses in Italy between 1999 and 2003 J Infect Dis 2005 192 8 1318 22 10.1086/444390 16170747

[B2] Koopmans M Wilbrink B Conyn M Natrop G van der Nat H Vennema H Meijer A van Steenbergen J Fouchier R Osterhaus A Bosman A Transmission of H7N7 avian influenza A virus to human beings during a large outbreak in commercial poultry farms in The Netherlands Lancet 2004 363 587 593 10.1016/S0140-6736(04)15589-X 14987882

[B3] Munasinghe S Brown G Pereira A Keeble B Nair P Sundkvist T Public health response to an avian influenza A (H5N1) poultry outbreak in Suffolk, United Kingdom, in November 2007 Euro Surveill 2008 13 5 pii=8027 http://www.eurosurveillance.org/ViewArticle.aspx?ArticleId=8027 18445374 10.2807/ese.13.05.08027-en

[B4] Editorial team Confirmed H5N1 avian influenza outbreak on a poultry farm in England, February 2007 Euro Surveill 2007 12 6 pii=3133 http://www.eurosurveillance.org/ViewArticle.aspx?ArticleId=3133 10.2807/esw.12.06.03133-en 17370950

[B5] Jefferson T Del Mar C Dooley L Ferroni E Al-Ansary LA Bahamdan SAS Bawazeer GA van Driel ML Foxlee R Rivetti A Physical interventions to interrupt or reduce the spread of respiratory viruses: systematic review BMJ 2009 339 b3675 http://www.bmj.com/cgi/reprint/339/sep21_1/b3675 10.1136/bmj.b3675 19773323 PMC2749164

[B6] Health Protection Agency Guidance for Health Protection Units on dealing with the human health implications of avian influenza in poultry and wild birds, 2007 http://www.hpa.org.uk/web/HPAwebFile/HPAweb_C/1194947343802

[B7] Influenza team (European Centre for Disease Prevention and Control) Outbreaks of highly pathogenic avian influenza (A/H5N1) in commercial poultry in Hungary and the UK - public health implications? Euro Surveill 2007 12 7 pii=3141 http://www.eurosurveillance.org/ViewArticle.aspx?ArticleId=3141 17370958

[B8] Health Protection Agency Occupational guidance for those responding to a suspected or confirmed avian influenza incident 2009 http://www.hpa.org.uk/web/HPAwebFile/HPAweb_C/1230540129063

[B9] Department for Environment Food and Rural Affairs Defra policy statement on the preferred options for the recovery/disposal of wastes likely to arise from an outbreak of avian influenza http://archive.defra.gov.uk/foodfarm/farmanimal/diseases/atoz/ai/documents/waste-disposal.pdf

[B10] European Centre for Disease Prevention and Control ECDC scientific advice - Who is at risk of getting HPAI? 2006 http://www.ecdc.europa.eu/en/publications/Publications/0605_TER_Avian_Influenza_Who_is_at_Risk.pdf

[B11] Avian influenza A (H1N1) infection in humans The writing committee of the World Health Organisation consultation on human influenza A/H5 N Engl J Med 2005 353 13 1374 85 16192482 10.1056/NEJMra052211

[B12] McQuiston JH Garber LP Porter-Spalding BA Hahn JW Pierson FW Wainwright SH Senne DA Brignole TJ Akey BL Holt TJ Evaluation of risk factors for the spread of low pathogenicity H7N2 avian influenza virus among commercial poultry farms J Am Vet Assoc 2005 226 5 767 72 10.2460/javma.2005.226.767 15776951

[B13] Bridges CB Lim W Hu-Primmer J Sims L Keiji Fukuda Mak KH Rowe T Thompson WW Conn L Xiuhua Lu Cox NJ Katz JM Risk of influenza A (H5N1) infection among poultry workers, Hong Kong, 1997-1998 J Infect Dis 2002 185 8 1005 10 10.1086/340044 11930308

[B14] Schultsz C Nguyen VD Hai le T Ha DQ Peiris JSM Lim W Garcia J-M Tho ND Lan NTH Tho HH Thao PX van Doorn HR Van Vinh Chau N Farrar J de Jong MD Prevalence of antibodies against avian influenza A(H5N1) virus among cullers and poultry workers in Ho Chi Minh City, 2005 PLoS ONE 2009 4 11 e7948 http://www.plosone.org/article/info:doi%2F10.1371%2Fjournal.pone.0007948 10.1371/journal.pone.0007948 19956765 PMC2776305

[B15] Health and Safety Executive Avoiding the risk of infection when working with poultry that is suspected of having H5 or H7 notifiable avian influenza http://www.hse.gov.uk/biosafety/diseases/aisuspected.pdf

[B16] Department for Environment Food and Rural Affairs Contingency plan for exotic animal diseases: framework response plan, 2009 http://www.defra.gov.uk/foodfarm/farmanimal/diseases/control/documents/framework-of-emergency-preparedness.pdf

[B17] Pollard SJ Hickman GA Irving P Hough RL Gauntlett DM Howson SF Hart A Gayford P Gent N Exposure assessment of carcass disposal options in the event of notifiable exotic animal disease: Application to Avian Influenza Virus Environ Sci Technol 2008 42 3145 3154 10.1021/es702918d 18522087

[B18] European Centre for Disease Prevention and Control ECDC Guidelines to minimise the risk of humans acquiring highly pathogenic avian influenza from exposure to infected birds or animals, 2005 http://ec.europa.eu/health/ph_threats/com/Influenza/ecdc_guidelines.pdf

[B19] Health and Safety Executive for Northern Ireland Bird flu (Avian Influenza) http://www.hseni.gov.uk/avian_flu_english.pdf

[B20] Department of Health The Influenza Immunisation Programme 2009/10 http://www.dh.gov.uk/en/Publicationsandstatistics/Lettersandcirculars/Professionalletters/Chiefmedicalofficerletters/DH_097550

[B21] Jefferson T Demichelli V Rivetti D Jones M Di Pietrantonj C Rivetti A Antivirals for Influenza in healthy adults: systematic review Lancet 2006 367 303 313 10.1016/S0140-6736(06)67970-1 16443037

[B22] Fiore AE Shay DK Broder K Iskander JK Uyeki TM Mootrey G Bresse JS Cox NJ Prevention and control of influenza. Recommendations of the Advisory Committee on Immunisation Practices MMWR 2008 57 RR07 1 60 18685555

[B23] European Centre for Disease Prevention and Control ECDC interim guidance. Public health use of influenza antivirals during influenza pandemics 2009 http://www.ecdc.europa.eu/en/publications/Publications/0907_GUI_Public_Health_use_of_Influenza_Antivirals_during_Influenza_Pandemic.pdf

